# The Rational Treatment of Common Symptoms among Infants—II

**Published:** 1911-04-22

**Authors:** Eric Pritchard

**Affiliations:** Senior Assistant Physician, Queen's Hospital for Children, etc.


### Hospital Clinics.

### THE RATIONAL TREATMENT OF COMMON SYMPTOMS AMONG INFANTS—II.

By ERIC PRITCHARD, M.D., Senior Assistant Physician, Queen's Hospital for Children, etc.

(Delivered at the Polyclinic, Chenies Street, W.C.)

Vomiting from ten to thirty minutes after taking of food, especially when the vomit contains solid curds, can be prevented by giving a few grains of citrate of soda at the time of, or shortly after, the feed. The citrate should be given freely, 5 grains to the ounce of milk, and then gradually reduced in quantity. If continuous large doses are given, constipation may result. If the vomit is strongly acid, 10 to 30 ♏ of milk magnesia is valuable, given just before the usual time of vomiting. Where the vomit is simply clear fluid, it is good to give undiluted milk with citrate of soda; the volume of food taken is then reduced. The treatment of vomiting occurring more than half an hour after feeding resolves itself into the treatment of dilated stomach. Washing out that viscus is not always easy in out-patient work; but you can lengthen the period between meals, give the meals in more concentrated form, and administer alkalies just before the vomiting usually occurs. The more ill a child is, the longer should be the intervals between the feeds. Vomiting may become an acquired habit, and then it is a good plan to exhaust the vomiting centre, as it were, by giving an emetic—Vinum ipecacuanhæ and ammonium carbonate—shortly before the meal. I have cured many habitual vomiters in this way.

### The Crying Child.

You may think continuous crying or screaming is a trivial symptom, but it is not trivial to the parent who has to be always with it. The late Dr. Cheadle used to say there are two causes of continuous crying in children: (1) cutting the eye teeth, (2) earache. The first does not come within the scope of this lecture, because the eye teeth are not cut before the twelfth month; on the other hand, I have nevertheless found earache exceedingly common in infants. Otitis may also be suspected when there is a slight pharyngeal cough, when glands can be felt in the neck, and when the infant pulls its ear. Then it is justifiable to instil opium into the external auditory meatus. I generally use 10 minims of tincture of opium and 10 grains of carbolic acid to the ounce of glycerine. Fits of screaming are often associated with disturbance of the motor mechanism of digestion. Of the six infants in this category, four were suffering from dysperistalsis due to mucous-colitis, one from earache, and one from laryngitis. Laryngitis in infants can be recognised by the hoarse character of the cry, and the painful cough. It is rapidly relieved by hot fomentations to the throat, and by expectorants. Many think that thirst is a common cause of crying, but that has not been my experience. Much might be said on the psychology of crying; but it is certain that the personality of some women has a baneful influence upon some infants, whereas other women have an undoubtedly soothing effect. It is astonishing how quickly infants find when crying pays them and when it does not, and they readily recognise when the contest is of no use.

### Coughs.

The commonest cough, at least in London, is the pharyngeal cough. Very slight irritation and congestion will cause it, and this reflex symptom in an infant is difficult to check. It may be a precursor of adenoids, Eustachian catarrh, and running ears. I have had many infants from one month old upwards who have had cough due to the early development of adenoids in the nasopharynx, operated upon, and the result has been excellent. For the infants at Marylebone, we supply, at small cost, a glass syringe and antiseptic of the following composition, as a mouth and nose wash:—

Lotion Thymolis.

| | |
|---|---|
| Acidi boraci | ℥ij. |
| Benzoic. acid | ʒj. |
| Thymol. | ʒj. |
| Eucalyptol | ʒss. |
| Glycerini | ℥viij. |
| Spirit rectificati | ℥ij. |
| Liquor cocci | ʒij. |
| Aquam ad | ℥xviij. |

It costs only about 1s. per pint, and is almost as good as glycothymoline. It is advisable to add the cochineal to colour it. Mothers must be taught to inject the liquid to the back of the nose, not in an upward direction, which is their tendency. A good plan is to paint the tonsils and pharynx with nitrate of silver, 10 grs. to the ounce.

### Bronchial Catarrh.

The next symptom is bronchial catarrh. I employ three stock mixtures for the three stages of the cough: (1) before there is a mucous secretion, (2) when the secretion is free, (3) when the secretion has become muco-purulent. These are for infants of three months old:—

No. 1.

| | |
|---|---|
| Potassii citratis | gr. ij. |
| Ammonium iodidi | gr. j. |
| Vinum ipecac (which can be increased to ♏x.) | ♏ij. |
| Tinc. Camph. Co. | ♏iij. |
| Syrup Aurantii | ♏x. |
| Aquam ad | ℥j. |

No. 2.

| | |
|---|---|
| Ammonii carbonatis | gr. j. |
| Oxymel scillæ | ♏v. |
| Vini ipecac. | ♏ij. |
| Syrup Tolutani | ♏v. |
| Aquam ad | ℥j. |

No 3.

| | |
|---|---|
| Ammonii carbonatis | gr. j. |
| Terebene | ♏ij. |
| Syrup Tolutani | ♏v. |
| Pulv. acaciæ | qs. |
| Aquam ad | ℥j. |

Whooping Cough.

Remember, young infants may have whooping cough when least suspected, because they often do not whoop. I think it is rare for children under three or four months of age to whoop. You miss this diagnosis by not making careful inquiry. In the whooping cough of infants the intervals between the paroxysms become longer as the disease progresses, and with this lengthening the attacks become more violent, but in ordinary bronchitis the intervals become shorter, and the attacks of cough are not so violent. The formula I employ for whooping cough is:—

| | |
|---|---|
| Potassii nitratis ... ... ... ... | ʒj. |
| Ammonii bromidi ... ... ... ... | ♏j. |
| Ammonii iodidi ... ... ... ... | ♏ij. |
| Tinct. belladonnæ ... ... ... ... | gr. j. |
| Vin. ipecac. ... ... ... ... | gr. j. |
| Aquam chloroformi ad... ... ... | gr. ij. |

As the case progresses I increase the belladonna, up to ♏v. or ♏vi.

Diarrhœa.

With regard to diarrhœa, there were only three cases of this disease in the series, owing to the season. In the summer the number of cases would be greater. For purposes of treatment, the cases of diarrhœa may be classified as follows: (1) Acute summer diarrhœa, (2) mucous colitis or enteritis, (3) lienteric diarrhœa. Summer diarrhœa must be drastically dealt with by suspending all food for twenty-four hours at least, and the colon should be washed out with hot water introduced at a low pressure by means of a soft rubber catheter. The temperature of the water should be 100° F., and the quantity not less than one pint. Small quantities of brandy and opium should be given by the mouth, in hot water if there is not much vomiting. Where the feet are particularly cold, give 10 minims of sweet spirit of nitre; this is also valuable for infants who have depressed fontanelles. It is irrational to give arrowroot or gruel; yet it is often given to infants who have not previously had starch. I never give arrowroot to children under twelve months old; and I have little faith in egg water in the severe cases. In most milder cases it is unnecessary to suspend breast-feeding, or to alter the bottle feeds. In mucocolis or enteritis, there are few drugs as good as castor oil. If the child suffers from tormina and cries much, I give large doses of glycerine of bismuth, sometimes with and sometimes without opium and chalk. In this stage all food must be given in a completely digested condition. A convenient way is to give Fairchild's predigested milk. For infants of three months old I order three ounces of that every three hours. The combination of threes makes it easier to remember. As the condition improves the food should be predigested less and less. It should be incubated for forty-eight minutes at the start, then forty, then thirty-eight, and so on, by which means the digestive processes are re-educated. In severe cases of this sort tonic treatment, by means of the hypophosphites and small doses of arsenic is indicated.

